# Hydroxypyridinones as a Very Promising Platform for Targeted Diagnostic and Therapeutic Radiopharmaceuticals

**DOI:** 10.3390/molecules26226997

**Published:** 2021-11-19

**Authors:** Xu Zhou, Linlin Dong, Langtao Shen

**Affiliations:** 1 HTA Co., Ltd., Beijing 102413, China; zhouxupku@126.com; 2 China Isotope & Radiation Corporation, Beijing 100089, China; donglinlin07@163.com; 3 National Isotope Center of Engineering and Technology, China Institute of Atomic Energy, Beijing 102413, China

**Keywords:** hydroxypyridinone, radiopharmaceutical, gallium-68, zirconium-89, thorium-227, theranostic, chelating agent, bifunctional chelators, siderophores, radiometal

## Abstract

Hydroxypyridinones (HOPOs) have been used in the chelation therapy of iron and actinide metals. Their application in metal-based radiopharmaceuticals has also been increasing in recent years. This review article focuses on how multidentate HOPOs can be used in targeted radiometal-based diagnostic and therapeutic radiopharmaceuticals. The general structure of radiometal-based targeted radiopharmaceuticals, a brief description of siderophores, the basic structure and properties of bidentate HOPO, some representative HOPO multidentate chelating agents, radiopharmaceuticals based on HOPO multidentate bifunctional chelators for gallium-68, thorium-227 and zirconium-89, as well as the future prospects of HOPO multidentate bifunctional chelators in other metal-based radiopharmaceuticals are described and discussed in turn. The HOPO metal-based radiopharmaceuticals that have shown good prospects in clinical and preclinical studies are gallium-68, thorium-227 and zirconium-89 radiopharmaceuticals. We expect HOPO multidentate bifunctional chelators to be a very promising platform for building novel targeted radiometal-based diagnostic and therapeutic radiopharmaceuticals.

## 1. Introduction

Nuclear medicine has become an important discipline in modern medicine. It can be used not only for diagnosis in oncology, cardiology, neurology, infectious and inflammatory diseases, but also for the treatment of malignant tumors and some benign diseases [1]. Nuclear medicine can be divided into diagnostic nuclear medicine and therapeutic nuclear medicine. Radiopharmaceuticals are essential in nuclear medicine. In the current diagnostic nuclear medicine, the most common diagnostic imaging modalities are SPECT/CT and PET/CT. In SPECT/CT, gamma-ray emitting radiopharmaceuticals are used; in PET/CT, positron-emitting radiopharmaceuticals are used. Radiopharmaceuticals that emit β particles, α particles, Auger electrons or conversion electrons are used in therapeutic nuclear medicine. In radiopharmaceuticals, both non-metallic radionuclides and metallic radionuclides can be used. Since the late 1950s, metallic radionuclides have been used in nuclear medicine for imaging and treatment. Among the metallic radionuclides, ^99m^Tc is the most widely used radionuclide. ^99m^Tc-based radiopharmaceuticals have always been the workhorse in nuclear medicine. More than 80% of all nuclear medicine procedures use ^99m^Tc-based radiopharmaceuticals [2], which are applied into about 25 million nuclear medicinal imaging procedures in the world every year. Since June 2016, FDA has approved five metal-based radiopharmaceuticals, including [^68^Ga]Ga-DOTATATE (in 2016), [^177^Lu]Lu-DOTATATE (in 2018), [^68^Ga]Ga-DOTATOC (in 2019), [^64^Cu]Cu-DOTATATE (in 2020) and [^68^Ga]Ga-PSMA-11 (in 2020). [^68^Ga]Ga-DOTATATE, [^68^Ga]Ga-DOTATOC and [^64^Cu]Cu-DOTATATE were used for the PET imaging diagnosis of somatostatin receptor positive neuroendocrine tumors (NETs). [^177^Lu]Lu-DOTATATE was indicated for the therapy of gastroenteropancreatic neuroendocrine tumors (GEP-NETs). [^68^Ga]Ga-PSMA-11 is a PET diagnostic imaging agent for prostate cancer. [^177^Lu]Lu-PSMA-617, another therapeutic radiopharmaceutical of prostate cancer, showed promising outcomes in a multicenter phase III randomized clinical trial (VISION) [3]. More and more big-name pharma companies (e.g., Novartis, AstraZeneca, et al. [4]) have entered the field of radiopharmaceutical. ^68^Ga/^177^Lu is a good-fit theranostic radionuclide pair. The successes of ^68^Ga/^177^Lu labeled radiopharmaceuticals in diagnoses and treatments of NETs and prostate cancer has inspired more research on other metal-based radiopharmaceuticals [5,6,7]. Among the metal-based radiopharmaceuticals, radiometal complexes account for the vast majority, except for a few simple inorganic compounds (such as [^89^Sr]SrCl_2_, [^223^Ra]RaCl_2_, etc.). In targeted metal-based radiopharmaceuticals, in addition to ligands that bind firmly to metallic nuclides, a biological targeting vector/molecule is usually contained. Therefore, when designing and synthesizing targeted metal-based radiopharmaceuticals, it is necessary to consider not only the selection of suitable metallic radionuclides, but also a ligand that matches the metallic nuclide. In addition, consideration must be taken not to affect the function of the biological targeting vector/molecule. The design and synthesis of targeted radiometal-based diagnostic and therapeutic radiopharmaceuticals have always been challenging tasks [8].

Hydroxypyridinones (HOPOs) are a class of privileged metal chelating agents with a wide range of uses. In recent years, the synthesis of hydroxypyridinones and their coordination chemistry in the field of metal (iron, aluminum, actinide, etc.) decorporation agents and gallium-68 PET radiopharmaceuticals have been well described and reviewed [9,10,11,12,13,14].

In this review article, we focus on how multidentate hydroxypyridinone (HOPO) chelating agents can be used in targeted radiometal-based diagnostic and therapeutic radiopharmaceuticals. The current status of these radiopharmaceuticals is also introduced. We will sequentially introduce the general structure of targeted metal-based radiopharmaceuticals, the basic structure and properties of hydroxypyridinones and some representative multidentate hydroxypyridinone chelators, the radiopharmaceuticals based on gallium-68, zirconium-89, thorium-227 and multidentate hydroxypyridinone chelators, and future prospects for the application of multidentate hydroxypyridinones in metal-based theranostic radiopharmaceuticals.

## 2. The General Structure of Targeted Metal-Based Radiopharmaceuticals

**Figure 1:**
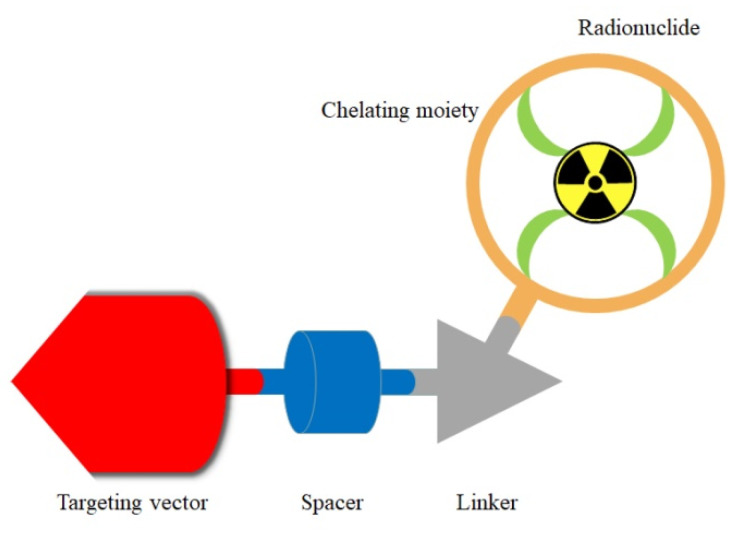
The general structure of targeted metal-based radiopharmaceuticals.

The general structure of targeted metal-based radiopharmaceuticals can be depicted in Figure 1. The structure of metal-based radiopharmaceuticals includes a metallic radionuclide, a chelating moiety, a targeting vector, a spacer and a linker [15].

A metallic radionuclide provides the function of radiation for imaging diagnosis or therapy. The bifunctional chelator is used to chelate a metallic radionuclide to form a metal chelate with thermodynamic stability and kinetic inertness. The targeting vector guides the accumulation of radiopharmaceuticals to the target of the disease area. The linker joins the radioactive part and targeting moieties. In some cases, the spacer is used to add distance between the two bulky molecules and avoid the interference of the targeting vector with the adjacent chelating portion. Another function of a spacer is to modulate the pharmacokinetic profile, such as to increase or decrease the lipophilicity of the whole radiopharmaceutical.

When designing novel metal radiopharmaceuticals, the choice of radionuclide is very important. Many factors should be considered in the selection of radiometallic nuclides. For example, is the radiopharmaceutical used for imaging diagnosis or therapy? If it is used for imaging diagnosis, we should further consider whether to use PET or SPECT imaging modality. For imaging diagnostic nuclides, we should also consider whether they have an appropriate physical half-life and energy. The physical half-life of the nuclide should match the biological half-life of the biological targeting vector. Radiometallic nuclides with short half-life are generally used for small molecule targeting vectors, while radiometallic nuclides with long half-life are used for targeting vectors of macromolecular (such as monoclonal antibodies, proteins, etc.) radiopharmaceuticals. For SPECT imaging, the energy of γ-rays between 100–250 keV is ideal. In PET imaging, radiometallic nuclides that emit low-energy positrons are the first choice. This is because low-energy positrons travel the shortest distance before generating positron annihilation. This means that metal-based radiopharmaceuticals that emit low-energy positrons can give high-resolution PET images. Table 1 shows the nuclear properties of some common diagnostic radionuclides used in nuclear medicine [5,6,16].

When talking about radio-therapic nuclides, we are mainly concerned with their decay properties, decay type and half-life. Due to the longer range of β^−^, nuclides that emit β^−^ particles are generally used for the treatment of larger tumors. For small tumors or metastases, it is advantageous to use alpha particles or Auger electron emitting nuclides. Similar to diagnostic radiopharmaceuticals, we also need to consider the half-life of the radionuclide. For radiopharmaceuticals with a small molecule as targeted vector, short half-life nuclides should be selected; for radiopharmaceuticals with macromolecules (such as monoclonal antibodies, proteins, etc.) as targeted vectors, radionuclides with long half-life are generally used. Table 2 shows some of the commonly used therapeutic radionuclides [5,6,16].

For common chelators, the properties of metallic radionuclides and the corresponding radiopharmaceuticals, readers could refer to review articles by Vermeulen, Kostelnik, Boros and Sneddon [5,6,8,17].

## 3. Siderophores, Bidentate Hydroxypyridinones and Multidentate Hydroxypyridinones

### 3.1. Siderophores, Bidentate Hydroxypyridinones

**Figure 2:**
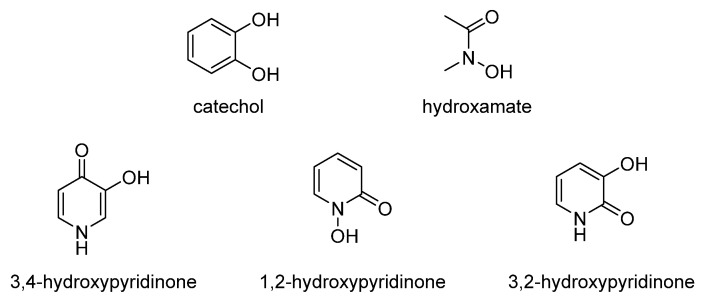
The structure of catechol, hydroxamate, 3,4-hydroxypyridinone, 1,2-hydroxypyridinone and 3,2-hydroxypyridinone.

Almost all living things need iron element. Microorganisms secrete low-molecular-weight compounds called siderophores to acquire iron from the environment. Neilands at Berkeley and Walter Keller-Schierlein of the ETH are pioneers in the study of siderophores [18]. They elucidated the chemical structure and function of some siderophores. Professor Raymond is the first chemist who studied the coordination chemistry of siderophores and the role of iron in siderophores [18]. So far, more than 500 siderophores have been discovered, isolated and characterized [19]. Large numbers of studies by several research groups have shown that the most common coordinating moieties chelated with ferric ion in siderophores are catecholate, hydroxamate, etc. [19,20,21,22]. However, hydroxypyridinone was also found in a few siderophores as ferric ion coordinating moiety [23,24]. The structures of bidentate hydroxypyridinones, catecholate and hydroxamate are shown in Figure 2.

Catechol and hydroxamate are two kinds of coordinating moieties that can bind ferric ions with high affinity. Compared with hydroxamate, catechol possesses a relatively higher affinity for ferric ion, though the chelation is sensitive to pH value [25].

Hydroxypyridinones are organic compounds with hydroxylic and ketonic oxygen atoms at appropriate positions on the 6-membered azaheterocycle. Bidentate hydroxypyridinones can be divided into 1-hydroxy-2-pyridone (1,2-HOPO or 1,2-HP), 3-hydroxy-2-pyridone (3,2-HOPO or 3,2-HP) and 3-hydroxy-4-pyridone (3,4-HOPO or 3,4-HP). 1,2-HOPO is actually a cyclic hydroxamic acid. Bidentate hydroxypyridinone is a potential coordinating group, and their anions form stable complexes with a series of metal ions [12]. The structures and properties of catechols, hydroxamates and common HOPOs are summarized in Table 3 [12].

Among these bidentate hydroxypyridinones, deferiprone is the most commonly studied and was approved by FDA for the treatment of thalassemia in 2011. Here we would like to highlight deferiprone as an example to introduce the basic physical and chemical properties. Deferiprone is a white crystalline solid which is soluble in water with a solubility of 16–18 mg/mL at 24 °C. It is stable in acid or basic solutions (pH < 1 or pH > 12) for over 2 years. In the aspect of acute toxicity, median lethal dose (LD50) range of deferiprone is about 600–700 mg/kg. No major toxic side effects were observed in animals at a dose of 200 mg/kg. For humans, there are no reported toxic side effects, except for certain adverse observations [26].

Generally bidentate hydroxypyridinones have two p*K*_a_ values, p*K*_a1_ and p*K*_a2_, and three cumulative constants (*β*_i_, i = 1–3) for equilibria concerning metal complex. *K*_ai_ could be calculated by [LH_i-1_][H]/[LH_i_] and *β*_i_ as [FeL_i_]/[Fe^3+^][L]^i^, where L denotes ligand. pFe^3+^ value was normally defined as –log[free Fe(III) in solution], when [ligand]_total_ = 10^−5^ M, [Fe^3+^]_total_ = 10^−6^ M and pH = 7.4. pFe^3+^ is introduced for comparison of chelating ability with Fe^3+^. The higher the pFe^3+^ value, the stronger the chelator’s Fe^3+^ binding affinity [12].

Among the three types of bidentate hydroxypyridinone, the hydroxyl group in 3,4-HP has the strongest alkalinity (p*K*_a__2_ = 9.5–9.9) [27], and has the strongest chelating ability with ferric ion at physiological pH. The affinity of 3,4-HP to hard Lewis acid metal ions such as Fe^3+^ and Al^3+^ is higher than that to biologically related divalent soft Lewis acid metal ions such as Fe^2+^, Zn^2+^ and Cu^2+^. In addition, 3,4-HP is quite stable under physiological conditions, and is easy to functionalize through the N atom on the heterocyclic ring or other positions on the ring. Therefore, 3,4-HP is one of the most commonly studied hydroxypyridinones so far.

The synthesis of bidentate hydroxypyridinones can be achieved with several common methods: 4-Substituted 1,2-HOPOs could be prepared from 2-chloropyridine-*N*-oxide; 6-Substituted 1,2-HOPOs could be prepared from 2,6-dibromopyridine; Substituted 3,2-HOPOs could be synthesized using 2,3-dihydroxypyridine as a starting material, and Mannich reaction could be used in the modification of 4, 5 and 6 positions of 3,2-HOPOs; N-Substituted 3,4-HOPOs could be achieved by double Michael addition; 2-Substituted 3,4-HOPOs could be prepared using Mannich reaction or aldol reaction; 5-Substituted 3,4-HOPOs could be obtained through the amino-methylation of 2-methyl-3,4-HOPOs, or could also be achieved through selectively brominated followed by substituted by methoxyl group; 6-Substituted 3,4-HOPOs could be prepared through the oxidation of kojic acid by Jones’ reagent, or could also be synthesized through the oxidation of 2-alkyl-3,4-HOPOs by Ag_2_O. For the detailed synthesis methods, please refer to the review article by Cilibrizzi et al. [12].

### 3.2. Multidentate Hydroxypyridinones

**Figure 3:**
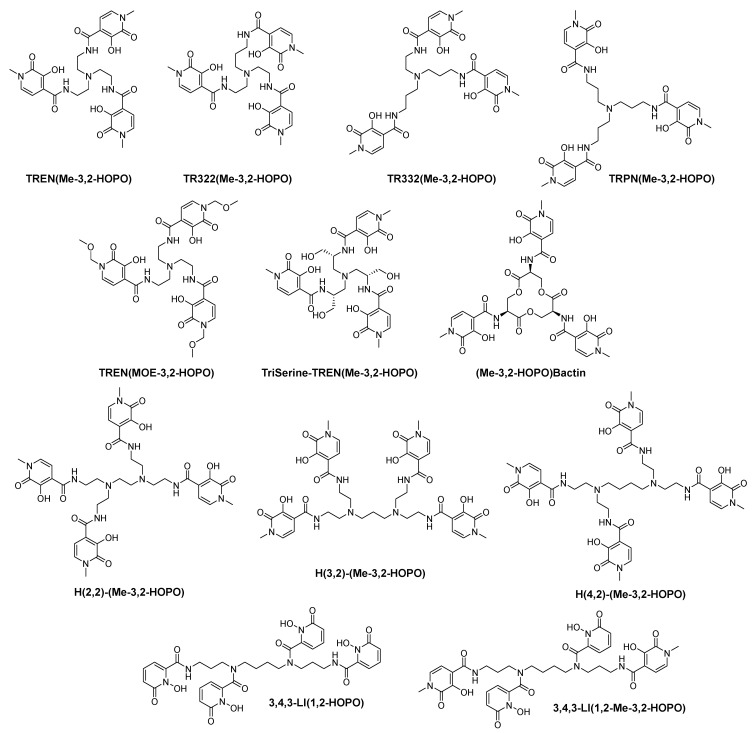
Some representative chelators of hexadentate and octa-dentate hydroxypyridinones from Raymond’s group.

Professor Raymond is a pioneer in the research of iron and actinide chelating agents. Taking tripodal enterobactin, cyclic ferrichrome and linear desferrioxamine B (DFO) as models, Raymond et al. designed and synthesized many artificial siderophores by using the approach of biomimetic chemistry. Among these siderophore analogs, catecholate, hydroxamate and HOPOs are used as coordinating groups [28]. In 1988, Raymond’s group first published the synthesis of a variety of multidentate hydroxypyridinones, such as 3,4-LIHOPO and 3,4,3-LIHOPO [29]. Some representative chelators of hexadentate and octa-dentate hydroxypyridinones from Raymond′s group are shown in Figure 3.

In Raymond′s group, 1,2-HOPO and 3,2-HOPO are often used to connect with different scaffolds (such as linear shape, cyclic shape, tripodal shape and “H” shape) to construct various hexadentate and octa-dentate chelators. They thoroughly studied the coordination chemistry of these multidentate HOPO chelators with ferric ions, gadolinium ions and actinides. The potential applications of their multidentate HOPO chelators as iron and actinide decorporation agents as well as MRI contrast agents based on gadolinium(III) ions were also investigated [28,30,31,32].

Raymond et al. developed a series of iron chelators named as TREN(Me-3,2-HOPO), TR322(Me-3,2-HOPO), TR332(Me-3,2-HOPO) and TRPN(Me-3,2-HOPO) [33]. The NMR data, crystal structure and solution chemistry of the ligands and the corresponding complexes showed the intramolecular hydrogen bonding. Among the four chelators, the TREN-capped ligand possesses the most acidic central amine. Besides, with the increase of the ligand size, the ferric complex stability shows a decreasing trend. As a result, TREN(Me-3,2-HOPO) is the strongest among these four chelators.

It is also worth mentioning that TREN(Me-3,2-HOPO) is a good chelator for gadolinium(III) ion [34].

Raymond’s group also investigated a series of TREN(Me-3,2-HOPO) derivatives, for example, TREN(MOE-3,2-HOPO), TriSerine-TREN(Me-3,2-HOPO) and (Me-3,2-HOPO)-Bactin.

To address the relatively low solubility of TREN(Me-3,2-HOPO) and its complexes in water, Raymond et al. designed and synthesized TREN(MOE-3,2-HOPO), which contains methoxyethyl groups. The Gd(III) complex of TREN-MOE-3,2-HOPO was prepared and the solubility of the complex in water was found to be on the order of 1 mM, which is about 10-fold compared with that of TREN-Me-3,2-HOPO [35].

After decorated with hydroxymethyl groups, TriSerine-TREN(Me-3,2-HOPO) was developed. TriSerine-TREN(Me-3,2-HOPO) shows increased water solubility, enhanced thermodynamic stability and higher relaxivity compared with TREN(Me-3,2-HOPO) [36].

(Me-3,2-HOPO)-Bactin was synthesized by Raymond et al. to address the low overall yield of the preparation of new synthetic analogs of enterobactin that retain the tri-lactone ring. This chelating agent could form a stable complex with ferric ion and the crystal structure of the ferric hopobactin was presented [37].

Apart from these hexadentate ligands, Raymond’s group also attempted to design and develop various octa-dentate ligands.

To obtain octa-dentate ligands that are less acidic and less toxic than 1,2-HOPO based molecules, “H” shaped ligands, (H(2,2)-(Me-3,2-HOPO), H(3,2)-(Me-3,2-HOPO) and H(4,2)-(Me-3,2-HOPO),) were studied by Raymond’s group. This series of ligands possess octa-dentate geometry and show an “H” shape. They were found to be highly effective for chelation of plutonium(IV) in vivo [38].

Except for “H” shaped molecules, the Raymond group also synthesized linear octa-dentate chelators, 3,4,3-LI(1,2-HOPO) and 3,4,3-LI(1,2-Me-3,2-HOPO). Compared with 3,4,3-LI(1,2-HOPO), 3,4,3-LI(1,2-Me-3,2-HOPO) possesses enhanced affinity towards actinide ions at physiological pH. This is due to the introduction of the Me-3,2-HOPO coordinating group, which is less acidic than 1,2-HOPO, into this octa-dentate ligand. Animal experiments showed that both ligands could remove plutonium from mice with low toxicity [29,39].

**Figure 4:**
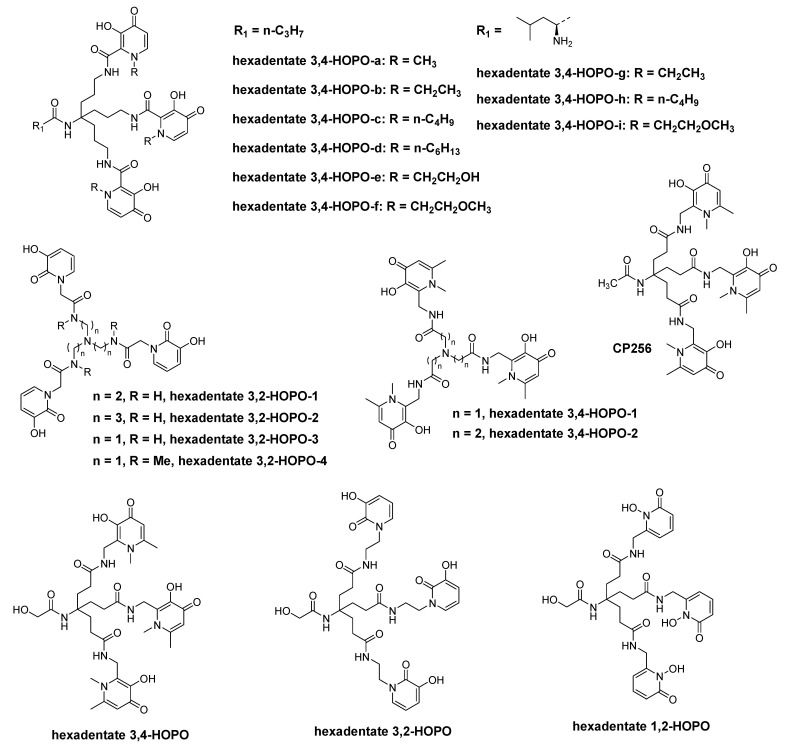
Some representative chelators of hexadentate hydroxypyridinones from Hider et al.

For a long time, Hider′s group has synthesized many HOPO iron chelators and studied their coordination chemistry and the effect of promoting iron excretion. In 1990, they synthesized two hexadentate hydroxypyridinone ligands with [NH_2_(CH_2_)_n_]_3_N (*n* = 2, 3) as the tripod and 3,2-HOPO as the coordinating group (hexadentate 3,2-HOPO-1 and hexadentate 3,2-HOPO-2 in Figure 4), and determined the p*K*_a_ of the ligands and the stability constants of the Fe(III) complex. They found that the stability constants of Fe(III) complexes with these two ligands are similar to that of DFO, and one of the hexadentate ligands can significantly enhance iron(III) excretion from hepatocytes and iron-overloaded mice [40].

In 1999, Hider et al. synthesized the same series of chelators, i.e., hexadentate 3,2-HOPO-3 and hexadentate 3,2-HOPO-4. Like their previous work, p*K*_a_ values and distribution coefficients of the ligands, and the stability constants of their iron(III) complexes were determined [41].

Hider and Zhou et al. prepared a series of hexadentate 3,4-HOPO based chelators with high affinity for ferric ion (hexadentate 3,4-HOPO-a to hexadentate 3,4-HOPO-i in Figure 4). Among these chelators, hexadentate 3,4-HOPO-a and hexadentate 3,4-HOPO-e showed the ability to inhibit the growth of both gram-positive and gram-negative bacteria, and they possess high pFe^3+^ values and high p*K*_1_ values. These chelators were found to exhibit the strongest inhibitory activity against staphylococcus aureus, bacillus subtilis, pseudomonas aeruginosa, and escherichia coli. This kind of chelators was found to possess potential application in the treatment of wound infections [42,43].

Hider and Zhou et al. also synthesized HOPO-based antimicrobial agents for the treatment of wound infections. Hexadentate 3,4-HOPO-1 and hexadentate 3,4-HOPO-2 were synthesized and both p*K*_a_ values of these chelators and their stability constants for the corresponding iron(III) complexes were evaluated. It showed that they possess high affinity for ferric ion, which could inhibit bacterial growth by Fe^3+^ starvation [44].

Hider and Zhou et al. synthesized three hydroxypyridinone based hexadentate ligands by conjugating the corresponding bidentate ligands (3-hydroxypyridin-4-one (3,4-HOPO), 3-hydroxypyridin-2-one (3,2-HOPO) and 1-hydroxypyridin-2-one (1,2-HOPO)) to a tripodal acid. The p*K*_a_ values and pFe^3+^ values of these ligands were investigated. Among these, hexadentate 3,4-HOPO possesses the highest pFe^3+^ value, which is inconsistent with the corresponding bidentate monomer 3,4-HOPO. As a result, hexadentate 3,4-HOPO holds the greatest potential as iron scavenging agent [45].

Figure 4 shows CP256 (i.e., THP-Ac) which demonstrated a relatively high affinity towards Fe^3+^ (lg *K*_1_ = 32.52, pFe^3+^ = 28.47), and the Fe(THP-Ac) complex is more stable than Fe(DFP)_3_. In 2011, Blower et al. found that the radiolabeling process of CP256 with Ga-68 could be completed within 5 min when the concentration of chelator CP256 is 10 μmol/L and pH = 6.5. Compared with HBED, the radiolabeling of CP256 could be accomplished under neutral pH, mild conditions and within a short period of time. When there was CP256 in ^68^Ga-transferrin solution, ^68^Ga-CP256 as a more stable complex could form immediately [46,47].

Santos′ group has done outstanding work in the field of metal chelating agents such as iron(III), aluminum(III) and gallium(III) [9,10,13,48]. They designed and synthesized a variety of hexadentate HOPO ligands and studied the chelation chemistry of these with metal ions.

**Figure 5:**
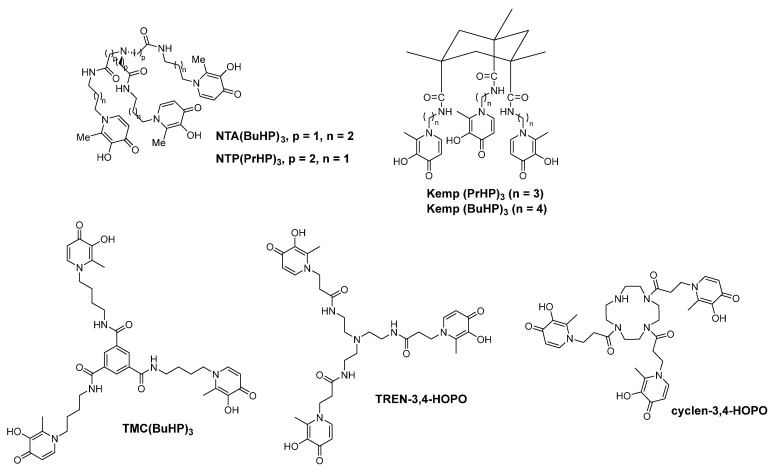
Some representative chelators of hexadentate hydroxypyridinones from Santos′ group and Shen′s group.

In 2009, Santos′ group synthesized two new tris-hydroxypyridinone based ligands (Kemp(PrHP)_3_ and Kemp(BuHP)_3_) (Figure 5). Among these, Kemp acid is used as a backbone, and 3,4-HP is the coordinating group. The stability and lipophilicity of the complexes with iron(III), aluminum(III) and gallium(III) ions and the ability of these ligands to promote gallium-67 excretion were investigated [49].

In 2010, Santos et al. developed two novel tripodal hexadentate 3,4-HP-based chelators NTA(BuHP)_3_ and NTP(PrHP)_3_ (Figure 5, NTA = nitrilotriacetic acid, NTP = nitrilotri-propionic acid, HP = hydroxypyridinone). These two chelators of ferric ion and aluminium ion are comprised of three 3,4-HP groups and a tripodal scaffold (NTA or NTP) moiety, with different positions of the carboxylic group. Both NTA(BuHP)_3_ and NTP(PrHP)_3_ showed high chelating capacity. As for the in vivo studies, these two chelators showed the same effects on the biodistribution and on the excretion of gallium-67 and high excretion rates, showing their potential application to remove iron or aluminum overload from the body. Compared with Kemp(PrHP)_3_ and Kemp(BuHP)_3_ developed by their own group, NTP(PrHP)_3_ and NTA(BuHP)_3_ showed higher chelating ability towards Fe^3+^ and Al^3+^ ions, respectively. This is largely due to the higher backbone flexibility of the nitrilo-based structures than the Kemp-based structures [10,49,50].

In 2011, Santos et al. studied the interaction between NTP(PrHP)_3_ and Ga^3+^, and the results showed that, compared with the commonly used chelating agents EDTA (pGa = 20.2), DTPA (pGa = 22.5), DOTA (pGa = 18.8) and transferrin (pGa = 20.3), the Ga/NTP(PrHP)_3_ complex has a higher pGa value (pGa = 27.5). This is because the three 3-hydroxy-4-pyridone chelating groups have a stronger affinity to Ga^3+^. The SPECT imaging and biodistribution results of ^67^Ga-NTP(PrHP)_3_ showed that, 24 h after intravenous injection, the radiolabel was mainly metabolized by the kidney, and no radioactivity was absorbed by other tissues. Because ^67^Ga-NTP(PrHP)_3_ has a strong absorption in bones, it possesses the potential to be used in the imaging of bone-related diseases. However, topological studies have shown that binuclear metal complex might exist, and there might be one of the three 3,4-HP groups in the dissociation state. Through 1:1 coordination, the stable metal complexes formed by NTP(PrHP)_3_ account for only 82%. In addition, since the fulcrum of the tripodal chelator is a nitrogen atom of a tertiary amine, it cannot be used as a bifunctional ligand for the coupling of targeted molecules [51].

Based on the experience in the field of artificial siderophores and chelating agents, and the increasing interest in metal-based radiopharmaceuticals [52,53,54,55,56,57,58], in 2010 Shen et al. developed a 3,4-HOPO based ligand, cyclen-3,4-HOPO. In this ligand, a cyclen was used as a tripodal scaffold [59,60]. In the same year, TREN-3,4-HOPO was also synthesized [60,61]. In TREN-3,4-HOPO, the tripodal scaffold is tris(2-aminoethyl)amine.

Recently, TMC(BuHP)_3_, a tris(3-hydroxy-4-pyridinone) hexadentate ligand based on 3,4-HOPO group and a backbone of 1,3,5-benzenetricarboxylic acid chloride, was prepared by Shen et al. The ferric complex formed between Fe(III) and TMC(BuHP)_3_ was confirmed [62,63].

In 1998, Martell et al. noticed that amide groups in hexadentate 3,2-HOPO-1 (CP130) and hexadentate 3,2-HOPO-2 might cause unfavorable conformational changes and thus lead to less stable complex. Therefore they used ether linkages to replace the amide groups and obtain the flexible TRISPYR. The new chelator showed a very high affinity for Fe(III). The results of molecular mechanics showed that TRISPYR possess an overall standard forcefield energy of 1.2 kcal mol^−^^1^, while the standard forcefield energy of hexadentate 3,2-HOPO-1 could not be minimized lower than 18.8 kcal mol^−^^1^ [64].

**Figure 6:**
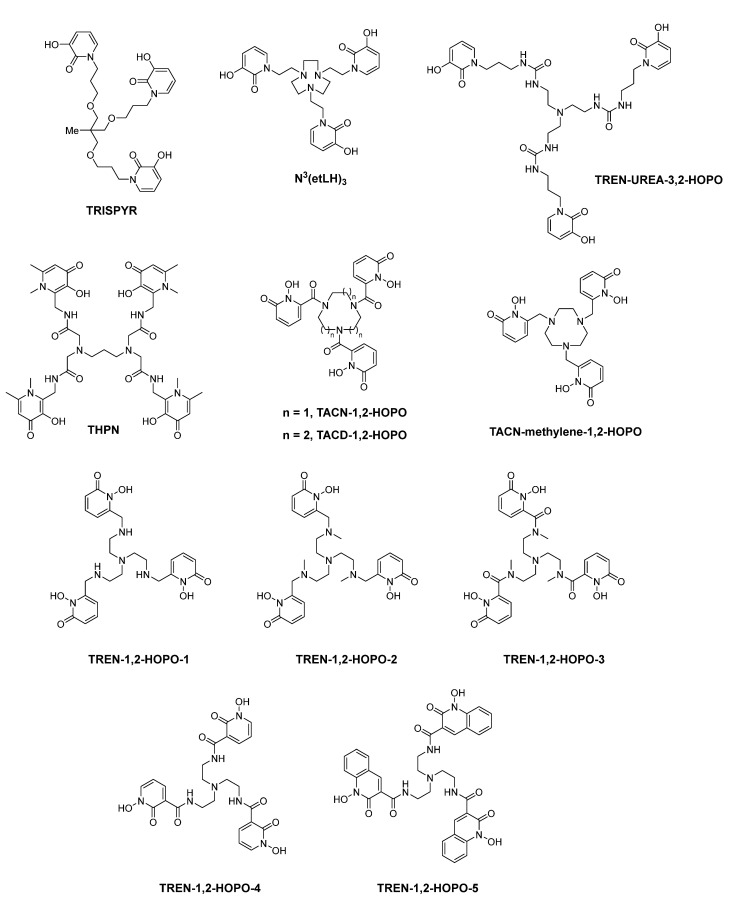
Some representative chelators of hexadentate hydroxypyridinones from other groups.

Crumbliss et al. prepared a hexadentate 3,2-HOPO based chelator named as N^3^(etLH)_3_ (Figure 6). N^3^(etLH)_3_ could form a sTable 1:1 complex with Fe(III) under the competition of EDTA and might be useful in iron chelation therapy [65].

Gopalan et al. developed a series of HOPO based linkers that could allow their attachment to various platforms. For example, when HOPO is functionalized with alkyne group, it could further take part in Sonogashira coupling or click chemistry. The author also developed chelators such as TREN-UREA-3,2-HOPO (Figure 6) and other chelators [66].

In 2017, Häfeli et al. developed a chelator with four 3-hydroxy-4-pyridinone (3,4-HOPO) coordinating groups, named as THPN. THPN was proved to quantitatively form a mononuclear complex with Zr^4+^ within 10 min at mild conditions (room temperature, pH is around 7.5) and relatively low concentrations. In vitro experiment showed that ^89^Zr-THPN is stable enough to resist the transchelation by free DFO (desferrioxamine B), even when the amount of DFO is 100 fold in excess. However, ^89^Zr-DFO could not withstand the challenge of THPN even when a tenth of THPN was present. In vivo experiments showed that in healthy mice ^89^Zr-THPN would undergo rapid renal excretion and no other organ uptake could be observed [67].

Tétard et al. prepared a series of triaza macrocyclic backbone based hexadentate 1,2-HOPO chelators, TACN-1,2-HOPO, TACD-1,2-HOPO and TACN-methylene-1,2-HOPO. TACN-methylene-1,2-HOPO is the first report of the use of this methylene group as linking strategy between 1,2-HOPO and the molecular scaffold. Experiments showed that the ferric complex of these chelators have a high stability and these chelators are effective against Gram negative and Gram positive bacteria [68].

Tétard et al. synthesized a series of hexadentate chelators combined with three 1,2-HOPO and a tris(2-aminoethyl)amine (TREN) core (TREN-1,2-HOPO-1, TREN-1,2-HOPO-2, TREN-1,2-HOPO-3, TREN-1,2-HOPO-4, TREN-1,2-HOPO-5). These chelators were prepared using secondary/tertiary amine/amide groups in order to evaluate the influence of size and flexibility of the linker. The investigations of physicochemical properties and biological activity showed that these chelators are powerful ferric chelators and are likely to inhibit bacterial growth by ferric ion starvation [69].

Stradling et al. investigated the excretion efficacy of HOPO analog (DFO-HOPO) towards ^238^Pu and ^241^Am. Although DFO-HOPO was ineffective for enhancing the elimination of inhaled or injected ^241^Am, it acted as the most effective treatment regimen for injected ^238^Pu. This work showed HOPO based chelators implied a possible application in the chelation of ^225^Ac, although there are some differences in the coordination chemistry between actinium and plutonium [70].

3,4,3-LI(1,2-HOPO) could bind firmly with Pu, Am, U, and Np and form excretable complexes with them at physiological pH. In 2015, Abergel et al. investigated the tissue and organ distribution of [^14^C]-3,4,3-LI(1,2-HOPO) in healthy male and female mice and rats. This investigation showed that [^14^C]-3,4,3-LI(1,2-HOPO) could quickly reach the systemic circulation, which exhibited that 3,4,3-LI(1,2-HOPO) should be a promising decorporation agent for actinides such as Pu, Am, U, and Np [71].

Recently, Abergel′s group used various methods to study the coordination chemistry of 3,4,3-LI(1,2-HOPO) with rare earth cations Sc^3+^ and Y^3+^. Their results showed that the binding of Sc^3+^ and Y^3+^ to 3,4,3-LI(1,2-HOPO) is strong, with high thermodynamic stability and rapid radiolabeling at room temperature, indicating that 3,4,3-LI(1,2-HOPO) may be a promising chelating agent of these two metals for in vivo diagnosis and treatment [72]. They also synthesized the octa-dentate ligands 3,4,3-LI(1,2-HOPO)_2_(CAM)_2_ and 3,4,3-LI(CAM)_2_(1,2-HOPO)_2_, and studied their thermodynamic and photophysical properties with Eu^3+^ and Tb^3+^. Both ligands showed high affinity for Ln(III) ion, and their stability constant of Eu(III) complex is greater than 10^29^ [73].

Phipps et al. synthesized 3,4,3-LI(1,2-HOPO) and investigated its chelating ability with ^47^Sc^3+^. They found that ^47^Sc-3,4,3-LI(1,2-HOPO) possesses high stability under various conditions. Comparisons between 3,4,3-LI(1,2-HOPO) and 3,3,3-LI(1,2-HOPO) are under investigation [74].

The above studies indicated that multidentate HOPO chelating agents may also be effective for rare earth Sc^3+^, Y^3+^ and lanthanide ions, although the application of HOPO bifunctional chelators in Sc^3+^, Y^3+^ and lanthanide radiopharmaceuticals has been less studied.

“Radiotheranostics” refers to the simultaneous imaging and treatment of target lesions using imaging diagnostic and therapeutic radiopharmaceuticals.

In theranostic radiopharmaceuticals, the radionuclide used for imaging diagnosis and therapy can be a combination of different element radionuclides, such as ^68^Ga and ^177^Lu, or a combination of different radionuclides of the same element, such as ^86^Y and ^90^Y. Rare earth metals and lanthanide metals provide us with a variety of possible theranostic radionuclide pairs [5,75,76].

At present, theranostic nuclide pairs that have received increasing attention are ^44/47^Sc, ^149/152/155/161^Tb and ^86/90^Y.

In nuclear medicine, scandium has two important nuclides, scandium-47 and scandium-44. Scandium-47 (t_1/2_ = 3.35 h) is a pure β^−^-emitter (E_β−avg_ = 162 keV, 100%). It can be used for the radiotherapy. Scandium-44 (t_1/2_ = 4.04 h, E_β+avg_ = 632 keV, 94%) is a promising PET radionuclides with high β^+^ branching ratio [77].

Terbium-149 (t_1/2_ = 4.12 h, E_α_ = 3970 keV, 17%) could be used in radiotherapy because it is an alpha-emitter. Terbium-152 (t_1/2_ = 17.50 h, E_β+avg_ = 1142 keV, 20%) decays by emitting positrons, so it is used for PET imaging. Terbium-155 (t_1/2_ = 5.32 d, E_γ_ = 87 and 105; I_γ_ = 32 and 25%, respectively) emits γ-radiation for SPECT imaging. Similar to ^177^Lu, terbium-161 (t_1/2_ = 6.89 d, E_γ_ = 26, 49, and 75 keV; I_γ_ = 23, 17, and 10%, respectively) emits low-energy β-particles and γ rays, and also contains a large number of Auger/conversion electrons (~12 e^−^/decay). Therefore, it can be used for therapeutic purposes [77].

Yttrium-86 (t_1/2_ = 14.7 h) can be used for PET imaging because it is a positron emitter. The therapeutic nuclide corresponding to yttrium-86 is yttrium-90. Yttrium-90 (t_1/2_ = 2.67 d) is a pure β^−^-emitting radionuclide. Due to the high energy of the emitted electrons (E_β-avg_ = 934 keV, 100%), it has a long range (about 11 mm) in soft tissues [5].

Scandium, terbium and yttrium have one factor in common, that is, under normal circumstances their stable oxidation state is +3, and the coordination number is generally higher than 8. In current radiopharmaceutical research, their bifunctional chelator is generally DOTA. Research on these radiometal ions and HOPO chelating agents is rarely reported. However, we believe that it is necessary to study the HOPO-based bifunctional octa-dentate chelator, chelation chemistry and their radiopharmaceuticals.

In addition, bismuth-213 and bismuth-212 are important therapeutic nuclides in the current TAT (targeted alpha therapy) with strong interest. Both bismuth-213 and bismuth-212 are alpha-emitter. The half-lives of Bi-213 and Bi-212 are 45.6 min and 60.6 min, respectively. The main chelating agents currently used for bismuth-213/212 radiopharmaceuticals are DOTA and DTPA [5]. Considering that Bi(III) is an intermediate Lewis acid, trivalent is the most common and stable oxidation state, and Bi(III) has a high coordination number and high affinity for multidentate ligands containing oxygen and nitrogen. We think it is meaningful to carry out research on the chelation chemistry and radiopharmaceuticals of HOPO-based multidentate ligands and Bi(III).

## 4. Diagnostic Radiopharmaceuticals Based on HOPOs

### 4.1. Radiopharmaceuticals Based on Ga-68 and Ga-67

#### 4.1.1. Nuclear Property of ^68^Ga/^67^Ga

The nuclear properties of commonly used gallium radionuclides are listed in Table 4.

In nuclear medicine, ^66^Ga, ^67^Ga and ^68^Ga are radionuclides that researchers would like to pay attention to. Among them, ^66^Ga and ^67^Ga are made by cyclotron, and ^68^Ga is usually produced with a ^68^Ge/^68^Ga generator although it could also be produced with a cyclotron [78].

While ^67^Ga decays to ^67^Zn by electron capture (100%), ^68^Ga decays to ^68^Zn by positron emission (89%) and electron capture (11%).

^67^Ga emits γ photons and can be used for SPECT imaging. As a result, ^67^Ga was once widely used in nuclear medicine, but it is not an ideal radionuclide for diagnostic imaging. The reason is that its γ photons abundance, suitable for imaging, is quite low compared to ^111^In, i.e., to achieve the same γ photon detection efficiency, the amount of ^67^Ga used must be three times that of ^111^In.

The half-life time of ^67^Ga is 78.3 h. The half-life time of ^68^Ga is 68 min and its parent isotope, ^68^Ge, possesses a longer half life time of 271 days. Therefore, the ^68^Ge/^68^Ga generator could be used in a relative long period of time. (Usually, a ^68^Ge/^68^Ga generator can be used for about one year) [5].

#### 4.1.2. Chemical Property of ^68^Ga/^67^Ga

Gallium is an element in group IIIA and the fourth period in the periodic table. The electronic configuration of gallium is [Ar]3d^10^4s^2^4p^1^. Such an electronic configuration renders gallium with smaller ionization energy. In aqueous solution, gallium ion exists in the +3 oxidation state. Under physiological conditions, Ga(III) could not be reduced or oxidized [78].

According to the hard-soft acid-base (HSAB) theory of Pearson, due to its high charge and small ion radius, Ga(III) belongs to the hard acids. Therefore, Ga(III) can interact with ionic, non-polar hard bases such as oxygen and nitrogen donor atoms, i.e., carboxylate, catecholate, hydroxamate, phosphonate, amine, etc., thus forming thermodynamically stable complexes.

When pH is between 3 and 7, Ga(III) undergoes a hydrolysis reaction to produce insoluble Ga(OH)_3_. When pH value is larger than 7, Ga(OH)_3_ would re-dissolve into Ga(OH)_4_^−^. When Ga(OH)_3_ or Ga(OH)_4_^−^ is formed, the kinetics of substitution reactions between multidentate ligands and these hydroxides are very slow. Therefore, the formation of Ga(OH)_3_ and Ga(OH)_4_^−^ must be avoided. Usually the radiolabeling of ^68^Ga should be carried out in the presence of weak ligands such as citrate, acetate and oxalate to avoid the formation of Ga(OH)_3_ [14].

#### 4.1.3. Common Bifunctional Chelators of Ga-68/Ga-67

The properties of Ga-68 in the aspect of solution chemistry and coordination chemistry have been discussed in the previous section. When designing a Ga(Ⅲ) bifunctional chelator, those properties together with the requirements for a bifunctional chelator in metal-based radiopharmaceuticals should be taken into consideration [11]. For example, Ga(Ⅲ) and high-spin Fe(Ⅲ) possess similar ionic radius (Ga(Ⅲ), 0.62 Å; high-spin Fe(Ⅲ), 0.65 Å), charge number and electronic configuration (no ligand field stabilization energy), leading to similarity in the aspect of coordination chemistry and in vivo properties. Because of this similarity, Ga(Ⅲ) could undergo ligand exchange reaction with transferrin in the body, resulting in the accumulation of radioactive Ga(Ⅲ) in the lung, liver and bone. Transferrin has two iron binding sites and these two sites have high affinity to Ga(Ⅲ). Under normal blood bicarbonate concentration, the stability constants of Ga(Ⅲ) with these two binding sites are lg *β*_1_ = 20.3, lg *β*_2_ = 39.6, respectively; while the stability constants of Fe(Ⅲ) with these two binding sites are lg *β*_1_ = 22.8, lg *β*_2_ = 44.3, respectively [79,80].

Therefore, the main requirement for the bifunctional chelator of Ga(III) is that the thermodynamic and kinetic stability of the metal complex should be good, so as to avoid the hydrolysis of Ga(Ⅲ) and the occurrence of a transchelation reaction from the ligand to the transferrin during clinical use.

**Figure 7:**
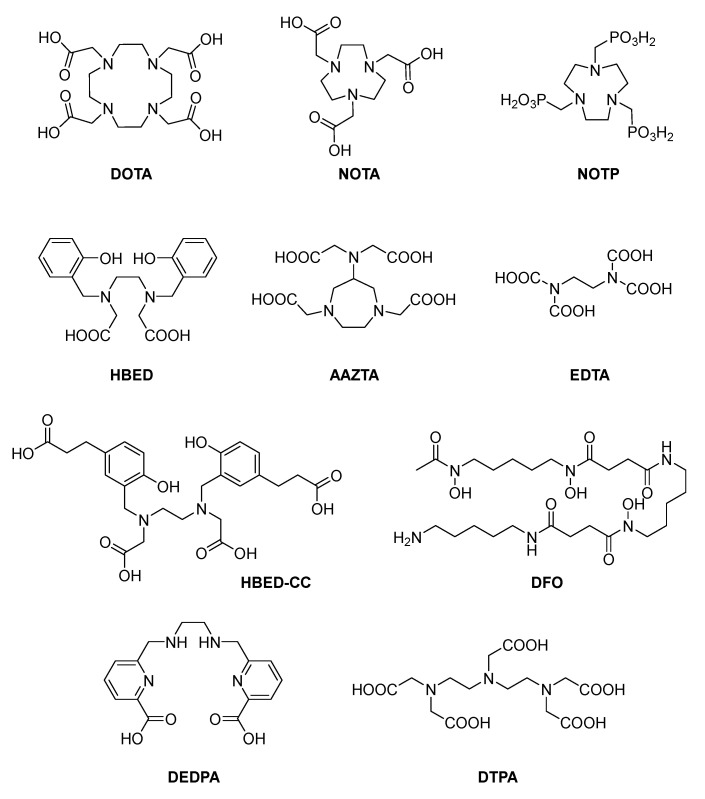
Common chelators of radiogallium.

After years of unremitting efforts, many Ga(III) chelators have been designed and synthesized. Representative common chelators are shown in Figure 7 [81,82,83].

According to structure, these chelators could be divided into cyclic chelators (DOTA, NOTA, NOTP, AAZTA) and acyclic chelators (HBED, HBED-CC, DFO, DEDPA, DTPA, EDTA). EDTA and DTPA could form 1:1 stable complexes with many metal ions and have good water solubility; however, they are not selective to Ga(Ⅲ) because they also chelate with divalent metal ions such as Zn^2+^ and Ca^2+^, leading to the loss of Zn^2+^ and Ca^2+^ ions in the body [27,84]. Besides, due to the concentration of Zn^2+^ and Ca^2+^ ions in the body being much higher than that of radioactive gallium(III) ion, these ions will also have a great impact on the stability of radioactive gallium(III)-DTPA (or EDTA) radiolabels. Therefore, these chelators have shown limited clinical applications.

The most commonly used metal chelator in nuclear medicine is DOTA [85], which could effectively chelate Ga(Ⅲ), but its chelating rate is slow. Therefore, when gallium-68 is labeled, it needs to be heated under acidic conditions (about 90 °C, 5–10 min), Moreover, the radiolabel of gallium often needs to be separated and purified (because the radiolabeling yield is less than 95%) [86]. NOTA could chelate gallium at room temperature, but the labeling time takes 15–60 min. Therefore, in order to increase the radiolabeling rate, in most cases the radiolabeling is carried out at around 95 °C [87]. Although AAZTA could radiolabel gallium at room temperature, its label is unstable in human serum proteins [88]. Under near-neutral conditions, the labeling result of NOTP with gallium is not ideal. When performing bioconjugation, its structure needs to be further modified [89]. There is currently still no report about ^68^Ga bioconjugate of NOTP. DFO is a versatile bifunctional chelator that can effectively radiolabel ^68^Ga at room temperature, but it is not suitable for use as a bifunctional chelator for ^67^Ga-labeled antibody radiopharmaceuticals because the radiolabel is not stable enough [90]. HBED-CC is the most commonly used derivative of HBED [91]. While gallium can be quickly radiolabeled by HBED-CC at room temperature, the HPLC results showed that isomers could be formed during the radiolabeling process, and the isomers possess different pharmacological properties, requiring heating to eliminate these isomers and increase the radiolabeling rate to more than 90%. At pH = 3–5, DEDPA could quickly and effectively label ^68^Ga at room temperature, but its radiolabel is not stable enough in human serum [92].

In summary, although the research on gallium chelators has made great progress, it is still a challenging task to design and synthesize better bifunctional chelators.

#### 4.1.4. HOPO-Based Bifunctional Chelators for Ga-68/Ga-67

HOPO-based bifunctional chelators showed potential application prospects in gallium-based radiopharmaceuticals. THP is one kind of new HOPO-containing bifunctional chelator for gallium-based radiopharmaceuticals.

**Figure 8:**
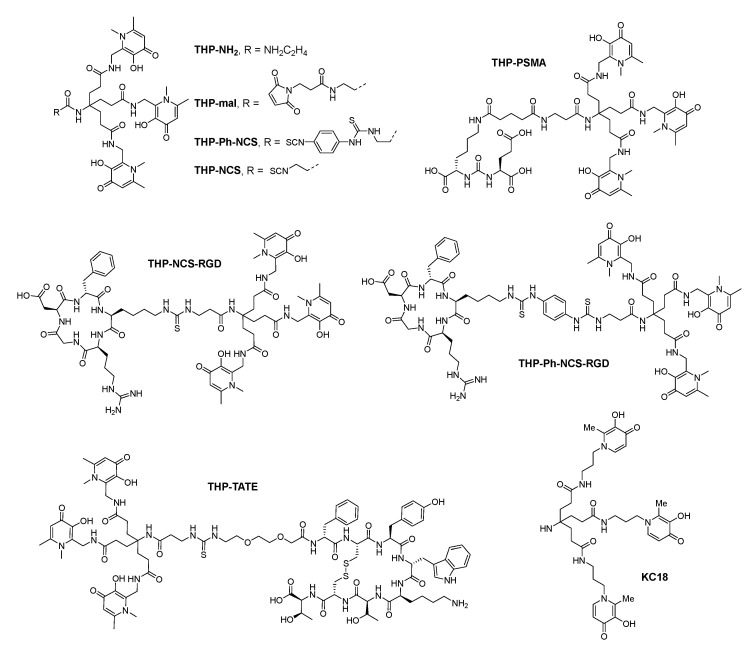
Structure of THP-PSMA, THP-NCS-RGD, THP-Ph-NCS-RGD, THP-TATE, KC18 and several derivatives of THP.

As shown in Figure 8, when R = NH_2_C_2_H_4_, THP-NH_2_ contains an amine group as the linker for bioconjugate. As for ^68^Ga-THP-mal-C2Ac (^68^Ga-YM-103-C2Ac), a PET imaging study was performed in a normal mouse. 90 min after injection, the complex was located almost exclusively in the kidney, except for some excretion to the bladder, while when uncomplexed ^68^Ga was injected, the distribution was spread throughout the whole body [47]. This shows that THP is an efficient chelator, with fast radiolabeling rate, mild condition and high radiochemical yield.

THP-Ph-NCS and THP-NCS were respectively linked to c(RGDfK) and formed THP-NCS-RGD and THP-Ph-NCS-RGD [93]. Both THP-NCS-RGD and THP-Ph-NCS-RGD could be radiolabeled completely within 5 min under the condition of room temperature, pH = 5.5–6.5 and low concentration, with a radiochemical yield of 95%-99%. Serum stability studies showed that no transchelation of ^68^Ga^3+^ from complexes to serum constituents occurred, neither in the case of ^68^Ga(THP-NCS-RGD) nor of ^68^Ga(THP-Ph-NCS-RGD). PET imaging of U87MG mouse and the biodistribution showed that both ^68^Ga(THP-NCS-RGD) and ^68^Ga(THP-Ph-NCS-RGD) could selectively target to α_v_β_3_ protein. Tumor uptake of ^68^Ga(THP-Ph-NCS-RGD) was a little higher than that of ^68^Ga(THP-NCS-RGD) at 1 h post-injection (2.86 ± 0.43 vs. 2.35 ± 0.06%ID g^−1^) and 2 h post-injection (3.32 ± 0.20 vs. 1.90 ± 0.21%ID g^−1^).

^68^Ga(DOTATATE) is currently a FDA approved radiopharmaceutical that is used in imaging of neuroendocrine tumors. However, the radiolabeling of DOTA needs to be performed under harsh conditions. To overcome this shortcoming, Ma et al. linked THP-NCS with TATE moiety, and performed the radiolabeling with ^68^Ga. Compared with DOTATATE, THP-TATE showed faster and milder radiolabeling conditions. The log *P*_OCT/PBS_ of ^68^Ga(THP-TATE) (−3.20 ± 0.09 (*n* = 6)) is higher than that of ^68^Ga(DOTATATE) (−3.69), indicating that ^68^Ga(THP-TATE) is significantly more lipophilic than ^68^Ga(DOTATATE). The PET imaging and biodistribution showed that ^68^Ga(THP-TATE) was successfully bound with SSTR2, which is almost the same with ^68^Ga(DOTATATE). However, ^68^Ga(THP-TATE) showed a comparatively longer retention in kidney although ^68^Ga(THP-TATE) and ^68^Ga(DOTATATE) had similar tumor uptake [94].

Nawaz et al. linked THP-mal with scFv (a single-chain variable fragment), a moiety which could target to PSMA. The radiolabel of ^68^Ga-THP-mal-J591c-scFv could be prepared with a high radiochemical yield (>97%) within 5 min and in mild conditions (room temperature, neutral pH). The radiolabel was stable in human serum. In cell binding experiments, ^68^Ga-THP-mal-J591c-scFv showed almost 10-fold higher binding to DU145-PSMA cells than to DU145 cells (PSMA negative). 3 h after injection, PET/CT imaging showed that ^68^Ga-THP-mal-J591c-scFv performed a quick blood clearance. The radiolabel has an uptake in DU145-PSMA tumor mouse, while in DU145 mouse (PSMA negative) there was no uptake of the radiolabel. Compared with other chelators such as DOTA and HBED-CC, THP-mal could chelate with ^68^Ga in a much milder and simpler way [95].

Young et al. further designed and synthesized a conjugate of THP with a small molecule inhibitor of PSMA, THP-PSMA, for the PET imaging diagnosis of prostate cancer [96]. Compared with ^68^Ga(DOTA-PSMA) and ^68^Ga(HBED-CC-PSMA) (^68^Ga-PSMA-11) [97,98], ^68^Ga-THP-PSMA is almost the same as these two radiopharmaceuticals in the aspect of tumor uptake, biodistribution, serum stability and pharmacokinetics, with a lower absorption in the spleen. The only difference is that the labeling conditions of ^68^Ga with THP-PSMA are more gentle, simple and easy to operate (ambient temperature, neutral pH, labeling time less than 5 min). The radiochemical yield is greater than 95%. Besides, the tripodal scaffold of THP limits the numbers of isomers occurrence, so there is no need to separate or purify after synthesis. As a preclinical evaluation of ^68^Ga-THP-PSMA, this investigation provides a basis for the kit-based preparation of ^68^Ga-THP-PSMA at room temperature.

Hofman et al. from the University of Melbourne also cooperated with Blower’s group to conduct a phase I clinical study of ^68^Ga-THP-PSMA. They screened 14 prostate cancer patients to study the safety and biodistribution of ^68^Ga-THP-PSMA. Eight patients in the experimental group were injected with ^68^Ga-THP-PSMA, and six patients in the control group were injected with ^68^Ga-PSMA-11. PET/CT results showed that for prostate cancers and metastatic tissues with overexpression of PSMA, the performance of ^68^Ga-THP-PSMA and ^68^Ga-PSMA-11 is almost the same. They both can quickly identify the cancer lesion, while ^68^Ga-THP-PSMA has a lower background absorption. The study proved that ^68^Ga-THP-PSMA is safe and effective. The success of ^68^Ga-THP-PSMA in clinical Phase I also indicates that ^68^Ga-THP-PSMA is expected to become another PET diagnostic imaging agent for prostate cancers after ^68^Ga-PSMA-11 [99].

In 2019, Santos et al. developed a 3,4-HOPO based bifunctional chelator named as KC18, with three HOPO units linked on a tripodal backbone. Molecular modelling studies based on Density Functional Theory (DFT) methods showed that the Fe^3+^-KC18 complex adopted a hexa-coordination and octahedral geometry. Through potentiometric and spectroscopic techniques, KC18 was proved to be a strong sequestering agent for ferric and aluminum ions without depletion of zinc ion. This result was also further evidenced by animal experiment with Ga-67 complex [100].

Until now, many HOPO-based hexadentate chelators have been synthesized. The high stability of these chelating agents and gallium(III) complexes has also been confirmed. However, since most are not bifunctional, they cannot be used for gallium-68 or other metal-based targeted radiopharmaceuticals. In 2006, Shen’s group started the design and synthesis of 3,4-HOPO-based bifunctional hexadentate chelators.

**Figure 9:**
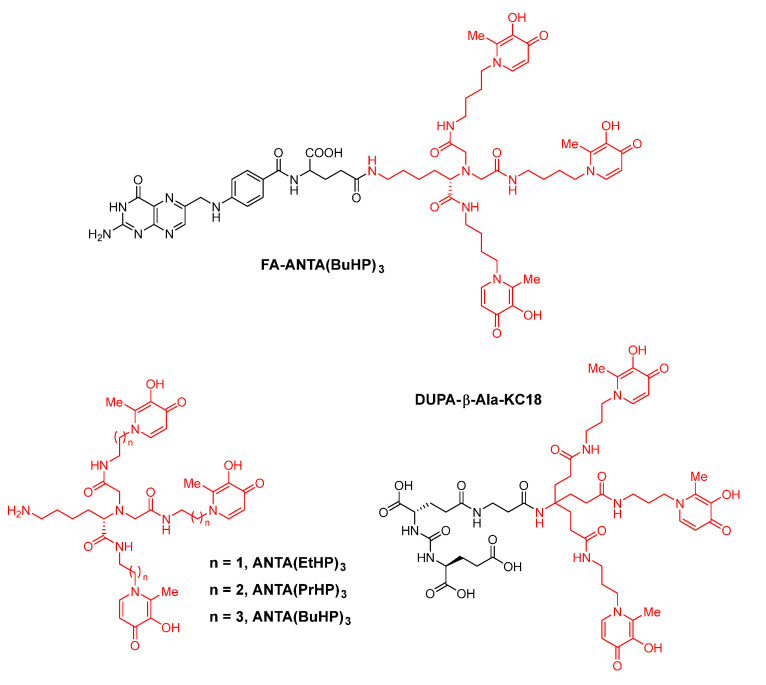
Tripodal bifunctional hexadentate chelators based on 3,4-HP from Shen’s group.

Firstly, Shen et al. used ANTA (*N*,*N*-bis(carboxymethyl)-l-lysine) as a tripodal scaffold and 3,4-HOPO as the coordinating group to synthesize a series of 3,4-HOPO-based bifunctional hexadentate chelators, such as ANTA(BuHP)_3_, ANTA(EtHP)_3_ and ANTA(PrHP)_3_ (Figure 9) [63,101,102,103,104]. Folate receptor (FR) is over-expressed in many tumors (such as ovarian cancer, cervical cancer, endometrial cancer, lung cancer, kidney cancer, breast cancer, colon cancer and brain cancer). Therefore, folate receptor is a target closely related to these tumors. ANTA(BuHP)_3_ has been successfully used to synthesize tumor-targeting gallium-68 precursor, FA-ANTA(BuHP)_3_ [105]. In FA-ANTA(BuHP)_3_, folic acid is a biological targeting vector.

In addition, Shen et al. also used di-tertbutyl 4-amino-4-[2-(tert-butoxy-carbonyl)ethyl]-heptane-dioate as another bifunctional tripodal scaffold [106] to synthesize a precursor of gallium-68 radiopharmaceutical in order to target prostate cancers. DUPA-β-Ala-KC18, as a novel potential precursor for prostate-specific membrane antigen (PSMA) targeted gallium-68 radiopharmaceutical, is a conjugate of DUPA and tris(hydroxypyridinone) chelator. Effective chelation between DUPA-β-Ala-KC18 and natural-abundance gallium(III) under room temperature and mild conditions were established by MALDI-TOF mass spectrometry. Further investigations are in progress [107].

### 4.2. Radiopharmaceuticals Based on Zr-89

Radiopharmaceuticals can be small molecule compounds or radiolabeled biological macromolecules, such as antibodies. Zevalin (^111^In- and ^90^Y-ibritumomab tiuxetan) is a monoclonal antibody drug that has been approved by the FDA for the diagnosis and treatment of non-Hodgkin’s lymphoma (NHL) [3]. Because monoclonal antibodies have high specificity for certain antigens, they can be used as therapeutic drugs for tumors and other diseases, such as the serious COVID-19 epidemic (coronavirus disease 2019). As of November 2020, 10 antibody therapies have been approved for the first time in the United States or the European Union [108]. In the past ten years, the development of antibody drug conjugates (ADC), multi-specific mAbs, immune checkpoint inhibitors, and mAb fragments such as single domain antibodies, nanobodies and antibody mimics, has become a trend [7].

For example, when the same cancer patients were treated using a therapeutic monoclonal antibody, some patients showed good therapeutic results, but other patients showed negative ones. ImmunoPET can be used to explain this difference in efficacy [109].

On the one hand, PET is an advanced medical imaging technique with high sensitivity and resolution; on the other hand, monoclonal antibodies bind to certain antigens with high specificity. ImmunoPET is an imaging technique that combines two advantages. In recent years, as more and more monoclonal antibody therapeutics have been approved and the availability of more long half-life radionuclides is achieved, immunoPET is developing rapidly, and its application fields are also expanding [110,111]. Among immunoPET imaging agents, ^89^Zr, ^64^Cu and ^68^Ga are the most commonly used radiometal nuclides. The biological half-life of an intact monoclonal antibody with a molecular weight of about 150 kDa is generally several days, so the biological half-life matches the physical half-life of ^89^Zr. ^89^Zr has received more and more attention in immunoPET [112]. Dilworth et al. gave an excellent review of the chemical properties of zirconium-89 used in PET imaging [113].

#### 4.2.1. Nuclear Property of ^89^Zr

The nuclear properties of ^89^Zr are listed in Table 5.

**Figure 10:**
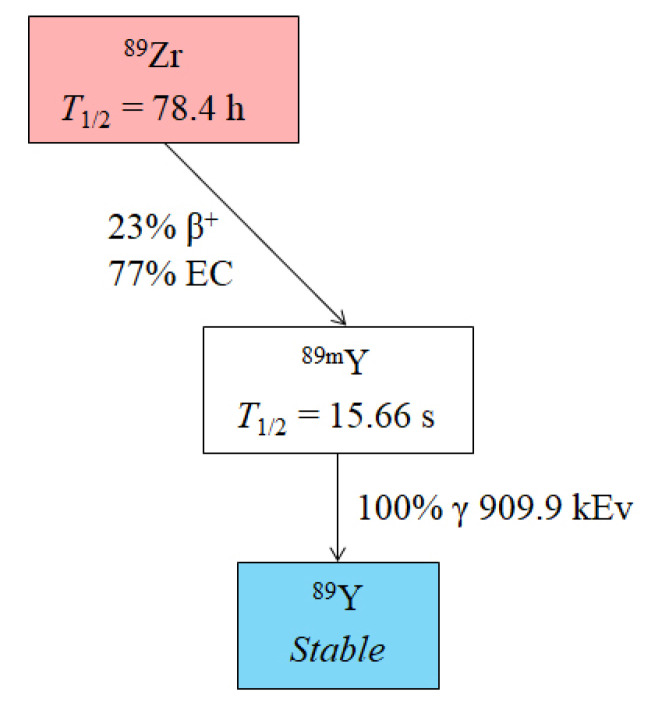
Decay process of ^89^Zr.

^89^Zr is produced by medical cyclotron through the reaction of ^89^Y(p,n)^89^Zr. The half-life time of ^89^Zr is 78.4 h, and it decays through the process of positron emission (23%) and electron capture (77%) (Figure 10) [114,115].

#### 4.2.2. Chemical Property of ^89^Zr

The element of zirconium is in group IVB and the fifth period in the periodic table. The electronic configuration of zirconium is [Kr]4d^2^5s^2^. In aqueous solution, zirconium ion has a valence of +4 and so Zr^4+^ possesses an electronic configuration of [Kr]4d^0^. Similar to gallium(III) ion, zirconium(IV) ion could be considered as hard Lewis acid due to its high charge and small ion radius. As a result, zirconium(IV) ion possesses a strong tendency to interact with hard Lewis bases such as oxygen donor atoms [114].

The most ideal coordination number of zirconium is 8, even though numbers 4–12 are possible.

#### 4.2.3. Common Bifunctional Chelators and HOPO-Based Bifunctional Chelators for ^89^Zr

The status of ^89^Zr bifunctional chelating agent has been introduced in several recent comprehensive review articles [6,7,113,115]. So far, the most used bifunctional chelating agents are iron chelator DFO (desferrioxamine B) and its derivatives. In this section we pay special attention to multidentate HOPO-based bifunctional chelating agents in ^89^Zr radiopharmaceuticals.

In 1992, Meijs et al. for the first time implied DFO as a Zr-89 chelator and found it to be extremely stable in human serum. As analogs of DFO, hydroxypyridinone-based chelators are attracting more and more interest from researchers around the world (Figure 7) [116].

**Figure 11:**
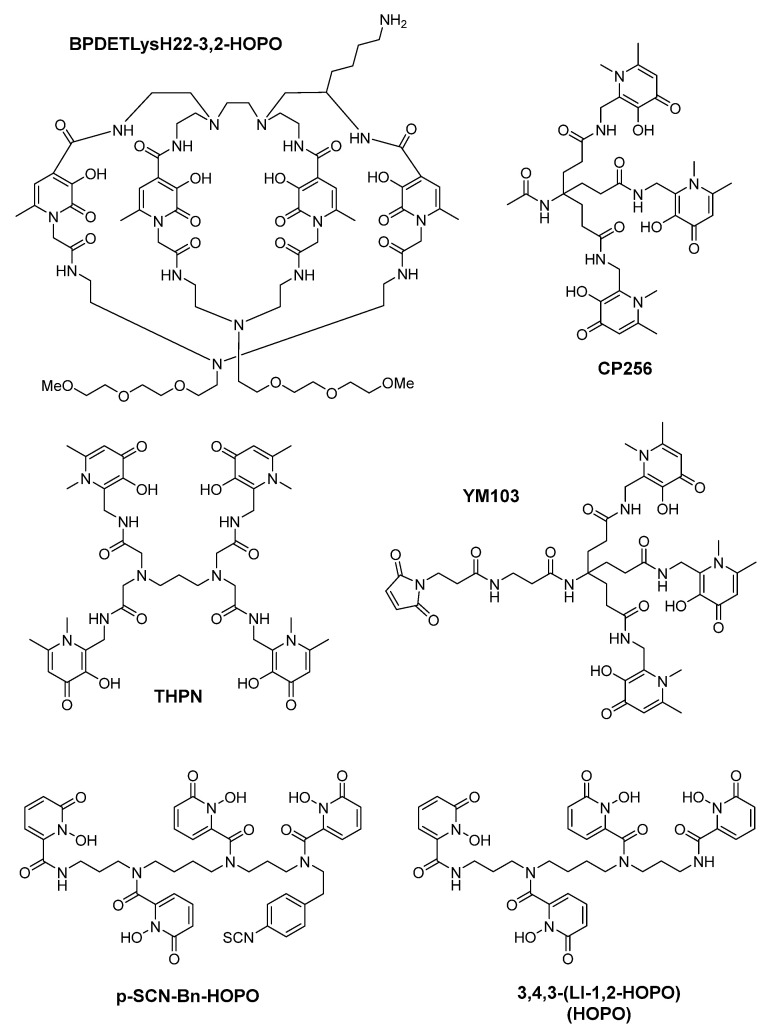
The structures of BPDETLysH22–3,2-HOPO, CP256, YM103, THPN, *p*-SCN-Bn-HOPO and 3,4,3-(LI-1,2-HOPO).

In 2014, Francesconi et al. investigated the ^89^Zr radiolabeling of 3,4,3-(LI-1,2-HOPO). 3,4,3-(LI-1,2-HOPO) (10 mM) could form a stable complex with ^89^Zr^4+^ at room temperature and pH 7. The radiolabel of ^89^Zr-3,4,3-(LI-1,2-HOPO) was stable under EDTA challenge. As a comparison, ^89^Zr-DFO suffered a transchelation process. In vivo experiments showed that in animal model ^89^Zr-3,4,3-(LI-1,2-HOPO) will go through renal excretion at early time points followed by hepatobiliary clearance at later time points [117]. DFT calculations on Zr^4+^-3,4,3-(LI-1,2-HOPO) and Zr^4+^-DFO showed that 3,3,3-(LI-1,2-HOPO) might bind more tightly to the Zr^4+^ than 3,4,3-(LI-1,2-HOPO) (Figure 11).

In 2015, Ma et al. evaluated a tripodal tris(hydroxypyridinone) chelator, CP256, together with its maleimide derivative, YM103, as the chelators of ^89^Zr^4+^. CP256 was chelated with Zr^4+^ forming a mononuclear complex which was confirmed by mass spectrum and NMR spectra. Radiolabeling of CP256 with ^89^Zr^4+^ was performed at pH 6.5 and in mild conditions. As a comparison, DFO was also used to chelate ^89^Zr^4+^. Both chelators showed quantitative coordination with ^89^Zr^4+^ at the concentration of 1 mM and 10 mM. However, low radiochemical yield was obtained in relative low concentrations. In competition studies, transmetallation of ^89^Zr^4+^ from CP256 to DFO could not be observed when DFO was added to a solution containing [^89^Zr(CP256)]^+^. However, dissociation of ^89^Zr^4+^ from DFO and coordination to CP256 could be measured by reverse phase radiochromatography when CP256 was added to [^89^Zr(DFO)]^+^ solution. In vivo experiments in mice indicate that the ^89^Zr-YM103-trastuzumab showed inferior stability compared with ^89^Zr-DFO-trastuzumab as it will release certain amounts of ^89^Zr^4+^, leading to bone uptake. This is largely due to hexadentate coordination geometry provided by three tris(hydroxypyridinone) groups [118].

In 2016, Marik et al. prepared a octa-dentate 3-hydroxypyridin-2-one (3,2-HOPO) based di-macrocyclic ligand named as BPDETLysH22–3,2-HOPO. This bifunctional chelating agent was radiolabeled with ^89^Zr and the radiolabeling could be completed under mild conditions within 15 min. The radiolabel was effective and stable in the presence of DTPA, while in serum a weak degradation could be observed. ^89^Zr-BPDETLysH22–3,2-HOPO demonstrates a LogP (partition coefficients) value of −1.53 ± 0.03, which is much more positive than that of ^89^Zr-DFO. In vivo experiment showed that elevated bone uptake could be observed, which might be due to a partial release of ^89^Zr^4+^ from the radiolabel. To evaluate the functionality of this bifunctional chelator, BPDETLysH22–3,2-HOPO was conjugated with HER2-specific trastuzumab and an isotypic anti-gD antibody, respectively. The radiolabeling yield of these two antibodies was in the range of 60% to 69%. The PET imaging of mouse model with HER2 positive ovarian carcinoma showed that tumor uptake of ^89^Zr-trastuzumab is 29.2 ± 12.9%ID/g, while that of ^89^Zr-gD is 6.0 ± 0.6%ID/g [119].

To simplify the synthetic route of 3,4,3-(LI-1,2-HOPO) derivative, Bhupathiraju et al. synthesized *p*-SCN-Bn-HOPO within four steps (formerly nine steps). Compared with the former work, in the new synthetic route preparative HPLC is only used in the last step. Besides, the overall yield of the preparation is increased from 1.4% to 14.3% [120].

In 2021, Lin et al. developed two HOPO based zirconium-89 antibody conjugates, [^89^Zr]Zr-3,2-HOPO-MSLN-mAb and [^89^Zr]Zr-DFO-MSLN-mAb. In in vitro experiments, [^89^Zr]Zr-3,2-HOPO-MSLN-mAb and [^89^Zr]Zr-DFO-MSLN-mAb both showed a high binding affinity for mesothelin (MSLN). In in vivo experiments, [^89^Zr]Zr-DFO-MSLN-mAb exhibited a higher tumor and lower femur uptake than [^89^Zr]Zr-3,2-HOPO-MSLN-mAb [121].

Until now, several Zr-89 based radiopharmaceuticals have been studied in clinical trials. For example, ^89^Zr-DFO-nimotuzumab could be used for diagnosis of lung cancer and colorectal cancer. This radiopharmaceutical is in phase II clinical trial (NCT04235114). ^89^Zr-DFO-huJ591 is also in phase II clinical trial and it is expected to detect prostate cancer for 18 years and older (NCT01543659). It is worth mentioning that ^89^Zr-TLX250 (^89^Zr-DFO-TFP-girentuximab), which is used to detect clear cell renal cell carcinoma, has been in phase III clinical trial (NCT03849118) [122].

## 5. Therapeutic Radiopharmaceuticals Based on HOPO Chelators

### 5.1. A Brief Introduction to Targeted Alpha Therapy (TAT)

Targeted alpha therapy (TAT) is defined as a therapeutic method that selectively delivers alpha particle-emitting radiopharmaceuticals to cancer cells and the tumor microenvironment to control tumors while minimizing toxicity [123,124].

The significant differences between alpha particles and beta particles in terms of mass, energy, and charge will lead to significantly different consequences when they interact with biological materials. The mass of the alpha particle is about 7300 times that of the beta particle. The travel distance of beta particles in biological tissues can reach 1.5–19 mm, while the travel distance of alpha particles is about 16–75 μm. Animal cells are eukaryotic cells with a diameter of 10–30 μm. This means that alpha particles can only pass through a few cells, while beta particles can pass through hundreds of cells [15].

Linear energy transfer (LET) refers to the energy that particles transfer to the affected substance within unit length. LET is expressed in keV/μm. High LET is a typical feature of high ionizing radiation and indicates dense deposition of energy, which causes great biological and chemical damage to biological tissues [125]. For the treatment of large tumors, particles that can produce long-range tissue effects are beneficial. However, it is increasingly recognized that long-range tissue effects are undesirable because it is directly related to off-target toxicity. The LET of particles and the range of action in biological tissues are related to radionuclides and are greatly affected by the type of decay and energy. The LET of β particles is about 0.2 keV/μm, and its action range is 1.5 to 19 mm, which is equivalent to the diameter of about 50 to 1000 cells; in contrast to β particles, the LET of α particles is as high as about 50 to 230 keV/μm. Its action range is about 16–75 μm, which is equivalent to the diameter of about 2–10 cells [123,125,126,127].

Different types of radiation cause different biological effects, which is defined as relative biological effectiveness (RBE). This is obtained by multiplying the dose by the weighting factor (WT). The larger the LET of a particle, the greater the biological effect produced, and therefore the higher the weighting factor assigned to it. The WT of a photon or electron is defined as 1, and the WT of a highly charged alpha particle is 20. Compared with beta particles, the RBE of alpha particles is greater [128]. Radiobiological cell survival studies have shown that the most effective LET for killing mammalian cells is around 100 keV/μm. At this optimal LET, the ionization event coincides with the diameter of the DNA double-strand (~2 nm), resulting in fatal DNA double-strand breaks in the cell [126]. When cells are irradiated with low-LET beta particles at a low dose rate, the cells can repair the damage during the irradiation. So it is difficult to kill the cells. Similarly, if the oxygen concentration is low or it is in an anaerobic state, such as in hypoxic and necrotic tissues, it is also very difficult to kill cells with radiation. However, alpha particles with high LET have strong cell killing efficiency, that is, their killing effect has nothing to do with radiation dose rate and oxygen concentration. Compared with alpha particle, 400 times the amount of beta particles are needed to kill a certain number of cells [15].

In short, when alpha particles are targeted to the disease site by appropriate methods, their huge energy is deposited at this local site, causing cell apoptosis through the break of the DNA double helix strand in the cell nucleus. The efficiency of alpha particles to kill cells has nothing to do with the oxygen concentration in the cells. Therefore, hypoxic tumor cells that are resistant in standard radiotherapy and chemotherapy are vulnerable to alpha particle therapy. Alpha particles cause direct damage to cells in a relatively small area. In addition, alpha particles also cause additional damage to adjacent cells through the bystander effect induced by radiation. However, compared with long-range beta particles, alpha particles have a smaller bystander effect and cause less indirect damage to normal cells in the adjacent tumor microenvironment.

### 5.2. HOPO-Based Bifunctional Chelators for Th-227

#### 5.2.1. Nuclear Property of ^227^Th

Thorium-227 is one of the alpha-emitting radionuclides. It can be used for TAT. The nuclear properties of ^227^Th are listed in Table 6.

**Figure 12:**
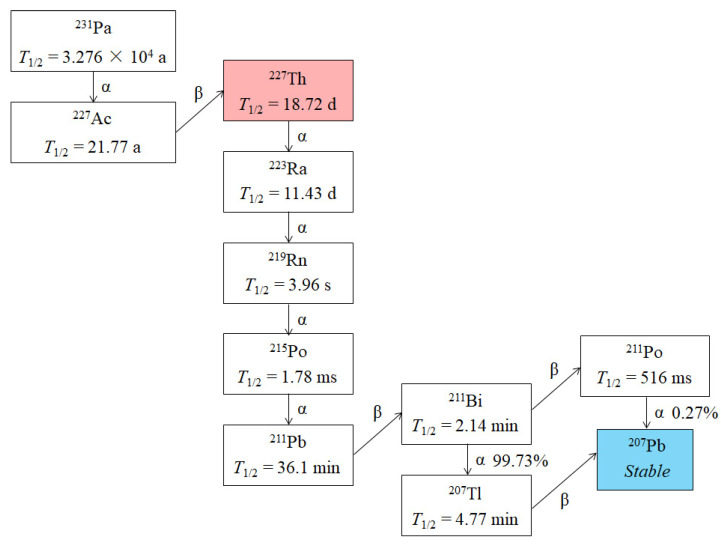
^213^Pa decay chains.

The decay process of ^227^Th is shown in Figure 12 [129] After five alpha decays, sTable ^207^Pb is finally formed from ^227^Th. ^227^Th first undergoes alpha decay, releasing energy of 5.9 MeV, forming daughter nuclide ^223^Ra with a half-life of 11.43 days. ^223^Ra undergoes alpha decay, releasing energy of 5.7 MeV, producing a daughter nuclide ^219^Rn with a half-life of 3.96 s. ^219^Rn undergoes alpha decay, releasing energy of 6.8 MeV, generating the daughter ^215^Po with a half-life of 1.78 ms. ^215^Po then undergoes an alpha decay process, releasing energy of 7.4 MeV, generating a daughter ^211^Pb with a half-life of 36.1 min. ^211^Pb is a β-emitter. After the decay, it produces a daughter ^211^Bi with a half-life of 2.14 min. The most important decay of ^211^Bi is through alpha decay, which releases energy of 6.6 MeV and generates ^207^Tl with a half-life of 4.77 min. ^207^Tl finally undergoes β-decay to produce sTable ^207^Pb. Another minor decay pathway of ^211^Bi is to produce the daughter ^211^Po with a half-life of 516 ms through β-decay process. Finally, ^211^Po undergoes α decay process, releasing energy of 7.4 MeV to produce the final stable product ^207^Pb [15].

#### 5.2.2. Chemical Property of ^227^Th

Thorium is the 90th element. It is located in group IIIB and the seventh period in the periodic table. It is the most abundant natural actinide element in the earth’s crust. The electronic configuration of thorium is [Rn]6d^2^7s^2^.

In aqueous solution, the only oxidation state of thorium is Th^4+^. At pH = 7.4 (close to physiological pH), thorium salt is hydrolyzed to form Th(OH)_4_ colloidal particles. In a 9-coordination environment, the ionic radius of Th(IV) is 1.09 Å, which is the largest and stable tetravalent metal ion. Since Th(IV) has the smallest ratio of charge to ion radius, it has poor hydrolysis compared with most metal ions, and can be studied in the pH up to about 4. Th(IV) in the aqueous solution undergoes multinuclear reaction, colloid formation and carbonate complexes formation. In addition, water-containing oxides or hydroxides of Th(IV) are difficult to dissolve [130]. Th(IV) ion belongs to the Lewis hard acids, which can form complexes with various ligands with different coordination numbers. The coordination numbers that have been found are 4–15. When Th(IV) is coordinated with small ligands, a very high coordination number can be achieved [126,131].

#### 5.2.3. Common Bifunctional Chelators and HOPO-Based Bifunctional Chelators for ^227^Th

In the past ten years, the most commonly investigated radiometals in TAT are ^225^Ac/^213^Bi, ^212^Pb/^212^Bi and ^227^Th [132,133]. Because ^227^Th has a long half-life, it is often used in monoclonal antibody-based radiopharmaceuticals. The current research focus of ^227^Th antibody-conjugated radiopharmaceuticals is in radiopharmaceuticals for breast cancer, lymphoma, kidney cancer, ovarian cancer, acute myeloid leukemia and prostate cancer [134,135].

In TAT, DOTA can be used as a bifunctional chelator for thorium, but the coordination kinetics are slow, requiring a higher labeling reaction temperature, which will lead to denaturation of the targeted antibody [134]. Therefore, it is necessary to develop a bifunctional chelator which could label thorium-227 with high efficiency and high yield under ambient temperature and mild conditions, and could make the radiolabel of thorium-227 with high purity and high stability. The development of octa-dentate HOPO chelating agents benefited from the research of thorium decorporation agents by Raymond′s group [28,136].

The HOPO chelating agents have a strong chelating ability not only towards Fe(III) and Ga(III) but also towards actinide ions. In fact they form complexes with high stability and low toxicity with actinides and they have been used as decorporation agents for this metal ions, as the case of thorium(IV) with octa-dentate HOPO compounds [28,136].

**Figure 13:**
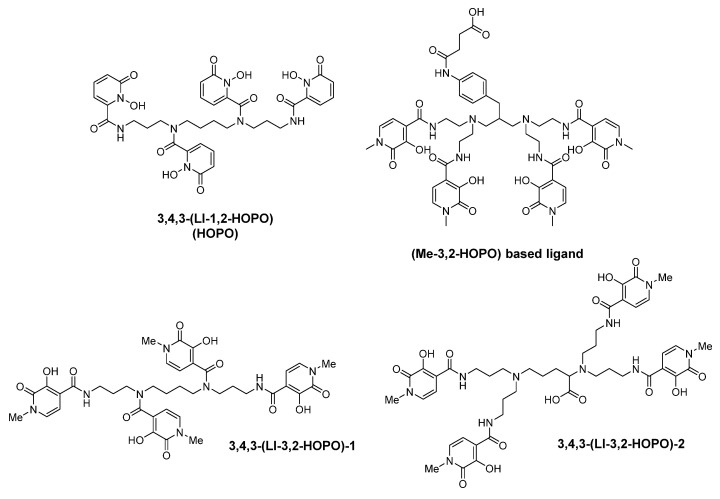
Structure of bifunctional HOPO chelators for ^227^Th.

As is shown in Figure 13, Abergel et al. investigated the solution coordination chemistry of lanthanide(IV) and actinide(IV) ions with 3,4,3-LI(1,2-HOPO). Using spectrophotometric pH-titrations, the authors determined the stability constants of [Th(IV)3,4,3-LI(1,2-HOPO)] (pTh^4+^ = 41.0(5)). This ligand was found to form strong and stable complexes with Th(IV) in aqueous solution (log *β*_110_ > 38.5) [137,138]. Except for 3,4,3-LI(1,2-HOPO), 5-LIO(Me-3,2-HOPO) was also evaluated and proved to be a strong chelator for Th(IV) (log *β*_120_ = 39.1(2)).

These works boost the development of 3-hydroxy-N-methyl-2-pyridinone (Me-3,2-HOPO)-based unit as the bifunctional chelator for thorium. Cuthbertson et al. developed a chelator (Figure 13) containing Me-3,2-HOPO as the coordinating group. This chelator could form stable complex with Th-227 within 20 min under room temperature (log *β*_110_ = 41.7(0.3)). In animal experiments, a hepatobiliary excretion route was indicated and bone uptake was low enough to prove the stability of the thorium complex in vivo [139,140].

The carboxylic acid group in this chelator could be used to conjugate with antibodies. For example, after conjugating with CD33 antibody (Lintuzumab), this Me-3,2-HOPO based chelator was found to possess the ability to radiolabel with Th-227 at neutral pH and ambient temperatures. This enabled the successful delivery of Th-227 to CD33 positive cells. In a biodistribution study, while an increase of radio activity over time in the tumor region could be observed, almost no accumulation of radio activity was present in other organs. In the anti-tumor activity experiment towards CD33-TTC, almost 83% of the animals were free of any palpable tumors at the end of the study. Furthermore, the tumor inhibition was influenced by the amount of Th-227. On November 26 2019, this drug (BAY1862864) completed its phase I clinical trial [141].

Cuthbertson et al. studied a CD70 targeted thorium-227 conjugate named as CD70-TTC, which is comprised of a CD70 targeting antibody, a 3,2-HOPO-based bifunctional chelator and alpha-emitting radionuclide thorium-227. This is till now the first report which investigated molecular targeting of CD70 expressing tumors using TAT. In vitro experiment showed that compared with the isotype control-TTC, the CD70-TTC possessed binding affinity and specific cytotoxicity to its target. A biodistribution study showed that compared with control-TTC, CD70-TTC retained significant uptake of ^227^Th by the tumor even seven days post injection [142].

Wilbur et al. investigated the chelation reactions between ^227^Th(IV) and two chelators with four coordinating groups of Me-3,2-HOPO. They found that these chelators could retain high chelation yields with thorium-227 within 2.5 h or less. The thorium complexes possess high stability in phosphate-buffered saline at ambient condition over a 6-day-period [143].

Till now, radium-223 is the only approved alpha-emitting radionuclide for the treatment of metastatic castration-resistant prostate cancer. As the progenitor nuclide of radium-223 (Figure 12), thorium-227 represents a promising alternative to radium-223, with the increasing emerging of efficient chelators for thorium. Recently Cuthbertson et al. gave a detailed review on 3,2-HOPO-based thorium-227 conjugates. These targeted thorium-227 conjugates, which combined 3,2-HOPO octa-dentate chelator with a range of targeting moieties, were supposed to be efficient to a series of hematological cancers [135].

## 6. Summary and Future Prospective

In metal-based radiopharmaceuticals, the concentrations of the metallic radionuclides and that of the chelators are very low. The metal-labeled radiopharmaceuticals are supposed to possess high thermodynamic and kinetic stability. Hydroxypyridinone based chelators possess low toxicity. Besides, they showed good selectivity and high stability towards some trivalent or tetravalent metal ions, such as gallium(III) ion, zirconium(IV) ion, thorium(IV) ion, etc.

^68^Ga-THP-PSMA, which is used for the diagnosis of prostate cancer by PET/CT imaging, has been undergoing a clinical phase II study (NCT03617588). The PSMA-targeted ^227^Th conjugate PSMA-TTC (BAY2315497) is a human anti-PSMA antibody linked to a chelator moiety (3,2-HOPO). This antibody-based therapeutic radiopharmaceutical is in a clinical phase I study (NCT03724747). The clinical phase I study of ^227^Th-Mesothelin-TTCs (BAY2287411) is also being carried out by Bayer (NCT03507452). This antibody-based radiopharmaceutical also uses 3,2-HOPO containing bifunctional octa-dentate chelator. Based on the promising preclinical results, ^89^Zr-HOPO-trastuzumab will be conducted in a clinical phase I-II study (NCT04757090). In short, HOPO-based multidentate ligands have shown clinical application prospects in the radiopharmaceuticals of gallium-68, thorium-227 and zirconium-89.

As mentioned above, although some HOPO-based radiopharmaceuticals of gallium-68, thorium-227 and zirconium-89 are in different phases of clinical trials, whether these radiopharmaceuticals could finally be approved for marketing still needs further investigation.

In addition to ^68^Ga, ^227^Th and ^89^Zr radiopharmaceuticals, could HOPO multidentate chelating agents be used in other metallic radiopharmaceuticals? This is an area worthy of further in-depth study.

In the current TAT, ^227^Th, ^225^Ac, ^213^Bi, ^212^Bi and ^149^Tb have become promising therapeutic radionuclides [144]. However, except for ^227^Th, the use of HOPO-based bifunctional chelating agents in radiopharmaceuticals of ^225^Ac, ^213^Bi, ^212^Bi and ^149^Tb has not been reported.

Regarding investigations of HOPO-based radiopharmaceuticals, we look forward to their deepening and expansion in the following directions:

(1) Through more extensive research, HOPO-based radiopharmaceuticals of ^68^Ga, ^227^Th and ^89^Zr should be promoted to market as soon as possible for the early diagnosis or treatment of various diseases, especially for cancers.

(2) TAT is expected to become one of the most effective approaches for cancer treatment. Various studies on HOPO-based radiopharmaceuticals of ^225^Ac, ^213^Bi, ^212^Bi and ^149^Tb should be carried out. The design and synthesis of HOPO-base bifunctional chelating agents for these metal radionuclides should be the first step.

Raymond et al. usually study lanthanide ions as suitable model analogues of actinide ions [28] when they design actinide decorporation agents. Both lanthanide and actinide metal ions belong to the hard Lewis acids. They have large ionic radii and flexible coordination geometries and prefer high coordination numbers (especially coordination numbers 8 and 9). Besides, they both prefer to interact with hard base donor atoms, such as oxygen or carboxylates, etc. Raymond et al. also conducted a series of studies on Gd-HOPO complexes as MRI contrast agents [30,31,32]. For example, the complex formed by H_3_-tren-Me-3,2-HOPO and gadolinium has a significantly high pGd value of 20.3 (ligand to metal concentration ratio of 10:1).

Among actinide decorporation agents and Gd-based MRI contrast agents, HOPO-based chelators showed strong chelating ability, which provides us with a basis in coordination chemistry for the further design of HOPO-based bifunctional chelating agents used in radiopharmaceuticals.

When designing HOPO-based bifunctional chelating agents for these metallic radionuclides, we must consider the different coordination chemistry properties of each metal nuclide. For example, although both Th and Ac are actinide elements, the common oxidation state of Th in solution is 4, while that of Ac is 3, and their coordination chemistry properties are quite different. In addition, for these metal ion bifunctional chelating agents, we not only hope that they have high chelating ability (which is consistent with the requirements of metal decorporation agents and MRI contrast agents), but also that they have rapid radiolabeling kinetics, in vivo stability, and a single configuration of the radiolabel. Therefore, the design and synthesis of bifunctional chelating agents containing multiple HOPO groups and a coupling group with biological targeting molecules is still challenging.

(3) The synthesis of HOPO-based bifunctional chelating agents often requires multiple steps. It is very important to simplify the synthesis steps and improve the overall synthesis yield. More efficient synthetic methods should be developed and employed. The improvement in synthetic methods could help increase the overall yield, which will be good for the development of radiopharmaceuticals from bench to bedside. Probably the molecular design and synthesis of bifunctional chelating agents, as well as the interactions between radiopharmaceuticals and biological targets, may be improved by virtue of artificial intelligence development [145,146].

With further expansion and in-depth study, we believe HOPO-based multidentate bifunctional chelators will become a very promising platform for novel targeted diagnostic and therapeutic radiopharmaceuticals.

## Figures and Tables

**Table 1:** Nuclear properties of some common diagnostic radionuclides.

Nuclide	*t*_1/2_	Type of Decay	Energy (keV)	Imaging Application
^99m^Tc	6.01 h	γ	140	SPECT
^123^I	13.2 h	γ EC ^1^/Auger	159 <5	SPECT
		
^131^I	8.0 d	γ β^−^	364 192 (mean)	SPECT
		
^67^Ga	78.3 h	γ	93 keV (38%); 184 keV (24%); 300 keV (22%)	SPECT
			
			
^111^In	2.80 d	γ	171 keV; 245 keV	SPECT
			
^201^Tl	3.042 d	γ	167	SPECT
^153^Sm	46.5 h	β^−^	807	SPECT
^18^F	110 min	β^+^	633	PET
		γ	140	
^68^Ga	68 min	γ	1080	PET
		β^+^	1899; 822	
^64^Cu	12.8 h	β^−^ β^+^	573 655	PET
		
^89^Zr	78.4 h	β^+^ γ	902 900	PET
		
^44^Sc	3.97 h	β^+^	632	PET
^86^Y	14.74 h	β^+^	3141	PET
		γ	1080	
^152^Tb	17.5 h	β^+^	1140	PET
^82^Rb	1.258 min	β^+^	4400	PET
^124^I	4.2 d	β^+^ Annihilation radiation	687 + 975 β^+^ (mean) 511 γ	PET
		

^1^ Electron capture decay.

**Table 2:** Nuclear properties of some common therapeutic radionuclides.

Nuclide	*t*_1/2_	Emission	Energy [MeV]_avg_
^131^I	8.0 d	β^−^	0.192 (mean)
^89^Sr	50.56 d	β^−^	0.587
^177^Lu	6.65 d	β^−^	0.134
^153^Sm	46.5 h	β^−^	0.807
^90^Y	64.0 h	β^−^	0.934
^223^Ra	11.4 d	α	5.8–7.5
		β^−^	0.173–0.495
^225^Ac	9.92 d	α	5.8–8.4
		β	0.198–0.660
^212^Bi	60.6 min	α	6.05
		β^−^	0.56–0.77
^213^Bi	45.6 min	β^−^	0.198–0.660
		α	5.9–8.4
^227^Th	18.9 d	α	5.8–7.5
		β^−^	0.173–0.495
^211^At	7.214 h	α	5.87 (41.8%)
^67^Ga	3.26 d	Auger e^−^	>0.0075
		CE	~0.08–0.09
^111^In	2.80 d	Auger e^−^	>0.019
		CE	~0.14–0.22
^47^Sc	3.35 d	β^−^	0.162
^149^Tb	4.1 h	α	3.97
		β^+^	0.725

**Table 3:** Structures and properties of the bidentate HOPOs and their analogs.

Ligand	Structure	p*K*_a1_	p*K*_a2_	log*β*_3_	pFe^3+^
*N*,*N*-Dimethyl-2,3-dihydroxybenzamide (DMB)	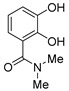	8.4	12.1	40.2	15
Acetohydroxamic acid	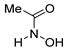	-	9.4	28.3	13
1-Hydroxypyridin-2-one	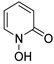	-	5.8	27	16
1-Methyl-3-hydroxypyridin-2-one	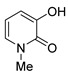	0.2	8.6	32	16
1,2-Dimethyl-3-hydroxypyridin-4-one (deferiprone)	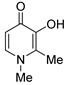	3.6	9.9	37.2	20

**Table 4:** Nuclear properties of commonly used gallium radionuclides.

Nuclear Properties	^66^Ga	^67^Ga	^68^Ga
Production Method	^66^Zn(p,n)^66^Ga	^68^Zn(p,2n)^67^Ga	^68^Ge/^68^Ga generator
Decay process	EC ^1^(43%)/β^+^(57%)→^66^Zn	EC(100%)→^67^Zn	EC(11%)/β^+^(89%)→^68^Zn
Half-life time	9.5 h	78.3 h	68 min
γ ray abundance	511 keV (114%)	93 keV (38%); 184 keV (24%); 300 keV (22%)	511 keV (178%)
		
		

^1^ Electron capture decay.

**Table 5:** Nuclear properties of ^89^Zr.

Nuclear Properties	^89^Zr
Production Method	^89^Y(p,n)^89^Zr
Decay process	EC(77%)/β^+^(23%)→^89m^Y
Half-life time	78.4 h
γ ray abundance	909 keV (99.9%); 1657 keV (0.1%); 1713 keV (0.8%)

**Table 6:** Nuclear properties of ^227^Th.

Nuclear Properties	^227^Th
Production Method	^227^Ac→^227^Th
Decay process	α→^223^Ra
Half-life time	18.72 d
α-emission energy (% abundance)	5709 keV (8.3%); 5713 keV (5.0%); 5757 keV (20.4%); 5978 keV (23.5%); 6038 keV (24.2%)


## Abbreviations

AAZTA	6-[Bis(carboxymethyl)amino]-1:4-bis(carboxymethyl)-6-methyl-1:4-diazepane
ADC	antibody drug conjugates
ANTA	*N*,*N*-Bis(carboxymethyl)-l-lysine
DEDPA	6,6′-((ethane-1,2-diylbis(azanediyl))bis(methylene))dipicolinic acid
DFO	desferrioxamine B
DFT	density functional theory
DOTA	1,4,7,10-tetraazacyclododecane-1,4,7,10-tetraacetic acid
DTPA	2,2′,2′′,2′′′-((((carboxymethyl)azanediyl)bis(ethane-2,1-diyl))bis(azanetriyl))tetraacetic acid
DUPA	2-[3-(1,3-dicarboxypropyl)-ureido]pentane-dioic acid
EDTA	ethylenediaminetetraacetic acid
FDA	US food & drug administration
FR	folate receptor
GEP-NETs	gastroenteropancreatic neuroendocrine tumors
HBED	bis(2-hydroxybenzyl)ethylene-diamine-diacetic acid
HBED-CC	3,3′-(((ethane-1,2-diylbis((carboxymethyl)azanediyl))bis(methylene))bis(4-hydroxy-3,1-phenylene))dipropionic acid
HOPO	hydroxypyridinone
HP	hydroxypyridinone
LET	linear energy transfer
NETs	neuroendocrine tumors
NHL	non-Hodgkin’s lymphoma
NOTA	1,4,7-triazacyclononane-1,4,7-triacetic acid
NOTP	((1,4,7-triazonane-1,4,7-triyl)tris(methylene))tris(phosphonic acid)
NTA	nitrilotriacetic acid
NTP	nitrilo-tripropionic acid
mAbs	monoclonal antibodies
PET	positron emission tomography
PSMA	prostate specific membrane antigen
RBE	relative biological effectiveness
RGD	cyclic RGDfK peptide
SPECT	single photon emission computed tomography
TACD	1,5,9-triazacyclododecane
TACN	1,4,7-triazacyclononane
TAT	targeted alpha therapy
TATE	octreotate
THP	tris(hydroxypyridinone)
THPN	1,3-propanediamine-*N*,*N*,*N*′,*N*′-tetrakis[(2-(aminomethyl)-3-hydroxy-1,6-dimethyl-4(1H)- pyridinone)acetamide]
TREN	tris(2-aminoethyl)amine
WT	weighting factor
